# Tracheal resection anastomosis for rare tracheal inflammatory lesions mimicking malignancy: report of 2 cases

**DOI:** 10.1186/s13019-024-02532-1

**Published:** 2024-02-03

**Authors:** Ahmed Musaad Abd-Elfattah, Alaa Gaafar, Hisham Atef Ebada, Mahmoud Seif-Elnasr, Ahmed Domain, Fedaey Ramadan Habaza, Khaled Zalata, Ali Tawfik

**Affiliations:** 1 Department of Otorhinolaryngology, Mansoura University, Mansoura, 35511 Egypt; 2 Alexandria University, Alexandria, Egypt

**Keywords:** Inflammatory myofibroblastoma, Inflammatory pseudotumor, Rosai–Dorfman disease, Tracheal, Resection anastomosis

## Abstract

**Background:**

Tumor-like lesions of the trachea are rare and challenging in diagnosis and management. Inflammatory myofibroblastoma, also known as Inflammatory pseudo tumors (IPTs), as well as Rosai Dorfman Disease (RDD) are inflammatory lesions that may involve the central airways with variable non-specific clinical features mimicking tumors.

**Case presentation:**

In this study 2 cases with tumor-like lesions are presented. One case with an inflammatory pseudotumor and the other one with Rosai–Dorfman disease affecting the upper trachea. Both cases were successfully managed with tracheal resection anastomosis.

**Conclusion:**

Tracheal Inflammatory myofibroblastoma, and Rosai–Dorfman diseases are rare tumor like lesions that present with upper airway obstruction. Despite being benign, these lesions may have features suggestive of malignancy, requiring prompt management. Complete surgical excision by segmental resection and primary anastomosis (if feasible) is the treatment of choice with an optimum outcome.

## Background

Tumor-like inflammatory lesions of the trachea as granulomatosis with polyangiitis, amyloidosis, and relapsing polychondritis, are rare and challenging in diagnosis and management. Diagnosis is often delayed due to nonspecific symptoms as cough, dyspnea, and stridor. They usually present in late stages unless incidentally discovered in an imaging study [1].

Inflammatory myofibroblastoma, also known as Inflammatory pseudo tumors (IPTs), as well as Rosai Dorfman Disease (RDD) are inflammatory lesions that may involve the central airways with variable non-specific clinical features mimicking tumors [2, 3].

In this study, we are reviewing our experience regarding tracheal resection anastomosis for those 2 aforementioned inflammatory lesions (IPT and RDD), involving the trachea. The outcomes of both cases as well as short- and long-term follow-up are highlighted.

## Case presentation

This is a combined work that was conducted in the Otorhinolaryngology Departments, Alexandria and Mansoura University Hospitals, Egypt. Informed written consents were obtained from the patients, and the study was approved from the local research board (IRB: R.23.04.2134).

### Case 1

A 32-year-old female presented to the outpatient clinic by slowly progressive respiratory distress in the last 3 months. Apart from hypothyroidism, the medical and surgical records were irrelevant.

The patient presented by severe biphasic stridor with suprasternal retractions. Indirect fiberoptic laryngoscopy revealed freely mobile vocal folds with reddish subglottic mass compromising about 80% of the airway lumen.

**Fig. 1:**
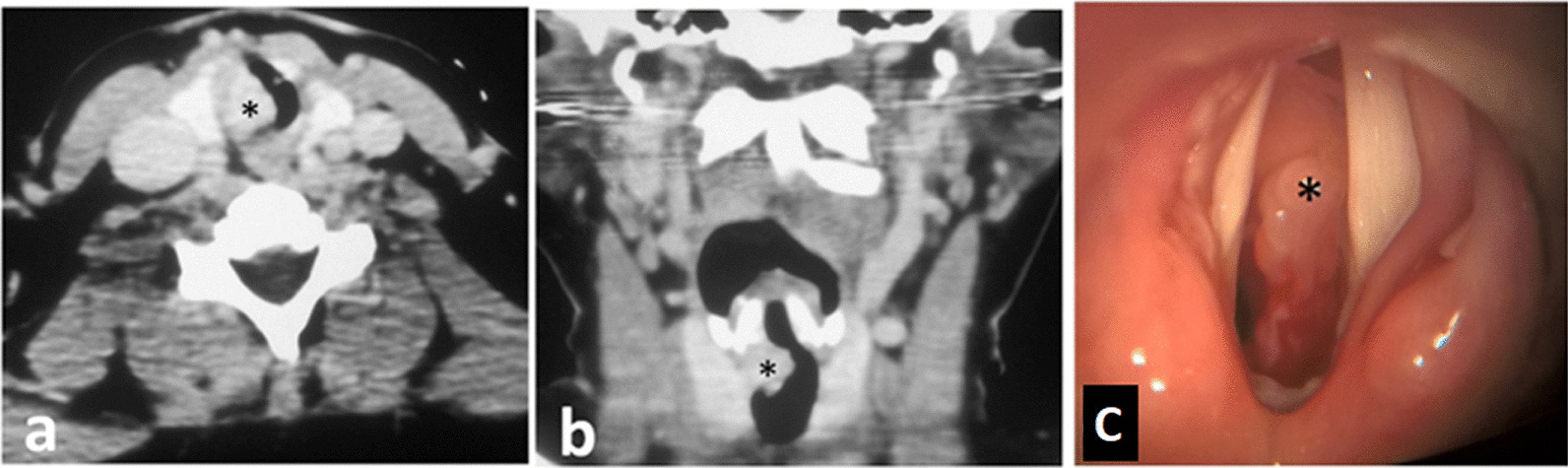
**a-**axial CT showing the soft tissue mass (*) occluding the tracheal lumen. **b** coronal CT showing the mass (*) extending from the lower border of the cricoid cartilage till the 3rd tracheal ring. **c** direct laryngoscopy showing soft tissue mass (*) below the vocal cords obstructing more than 80% of the airway lumen

CT scan with contrast revealed a sessile enhancing soft tissue mass (20 × 11 mm) involving the upper part of the cervical trachea encroaching upon subglottic region. No definite cartilage erosion was detected (Fig. 1a, b).

A decision of tracheostomy was made to secure the airway followed by direct laryngoscopy which revealed soft tissue fragile mass with bleeding on touch (Fig. 1c) and biopsy was obtained.

Pathology showed ulcerating respiratory and squamous epithelium encroached upon by a proliferative growth formed of spindle myofibroblastic cells forming interlacing and fascicular pattern. The stromal cells have bland looking nuclei with absent mitosis and admixed with chronic mononuclear inflammatory cells mainly lymphocytes and plasma cells. Immunohistochemical analysis revealed focal cytoplasmic positivity for smooth muscle actin and ALK (Anaplastic Lymphoma Kinase). The diagnosis of IPT was made.

This was followed by CO2 laser endoscopy with the purpose of debulking and curative outcome. Tracheotomy decannulation was successful after 5 days. However, recurrence was reported 6 months later. Another session of laser endoscopy was done which was also followed by recurrence within 4 months. For the aim of definitive treatment, the decision of complete excision was eventually made.

This was achieved by single stage partial cricotracheal resection anastomosis. Resection of the cricoid arch with the first 2 tracheal rings with thyrotracheal anastomosis was performed. The length of the resected segment was 2.6 cm.

Immediate postoperative extubation was done. With no reported intraoperative or postoperative complications. Follow up period was 3 years, and the patient was symptom free and returned to their usual daily activities within 3 weeks.

### Case 2

A 62-year-old male patient, presented to emergency unit with gradually progressive biphasic stridor over the last 2 month. Flexible transnasal laryngeoscopic examination revealed freely mobile vocal folds and an upper tracheal reddish mass.

**Fig. 2:**
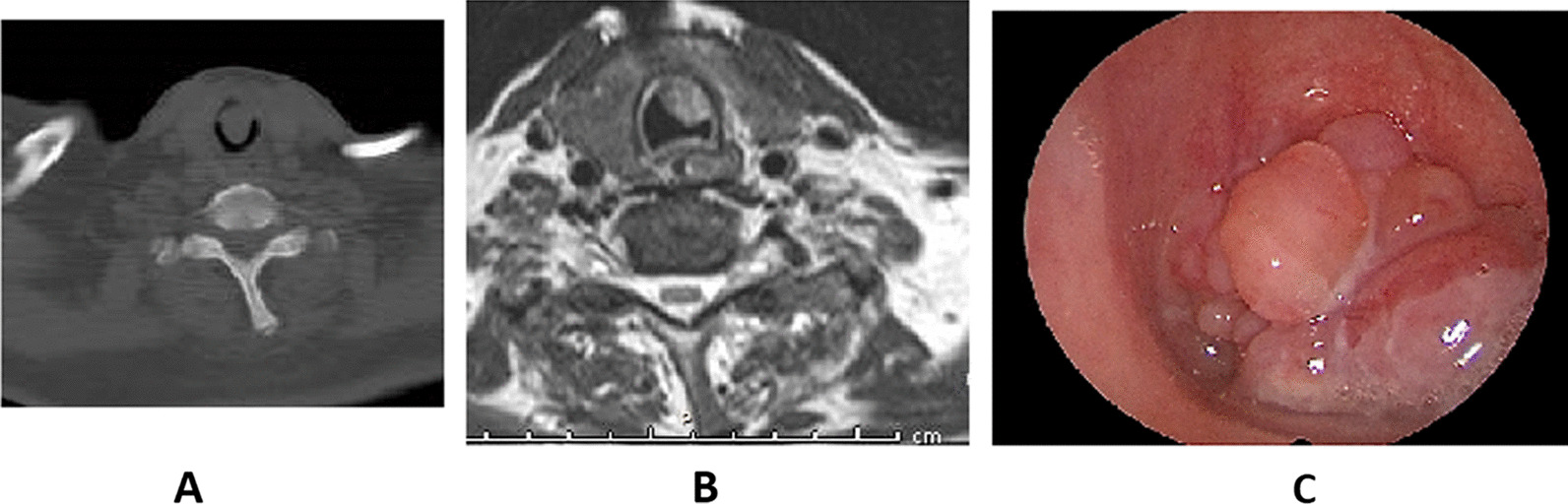
**a** CT scan showing the mass in the subglottic region and upper trachea. **b** MRI showing the mass without definite extra tracheal extension. **c** Direct laryngoscopy shows a lobulated mass in the upper trachea, occluding entire airway

Emergency tracheotomy under local anesthesia was made, followed by laryngotracheoscopy to obtain tissue for histopathology. A reddish lobulated mass, with smooth surface was detected occluding almost 75% of the tracheal lumen (Fig. 2c).

CT scan and MRI revealed an enhancing soft tissue mass (2 × 1.5 cm) in the upper 2 tracheal rings, with minimal cartilage invasion and without definite extra tracheal extension (Fig. 2a, b).

Pathology revealed emperipolesis and dense lymphohistiocytic infiltration with scattered large, atypical cells. Examination of slides stained by CD68 showed positive reaction of the large cells. Slides stained by S-100 were also positive. On the other hand, slides stained with CK revealed negative reaction. Final diagnosis of sinus hisotcytosis (Rosai–Dorfmann disease) was confirmed. After reviewing the literature and consultation of the multidisciplinary team, the decision of tracheal resection anastomosis was suggested. Resection of the first 3 rings was performed cricotracheal anastomosis. The resected segment was 2.3 cm in length.

The patient was immediately extubated at the end of the procedure. The postoperative course was smooth and uneventful. The patient was symptom free, and no recurrence was reported during the follow up period of 4 years.

## Discussion

Inflammatory pseudotumors (IPTs) as well as Rosai Dorfman disease are inflammatory lesions that can involve the trachea with a variable clinical course that may mimic malignant lesions [2, 4].

Inflammatory pseudo-tumors (IPTs) are uncommon solid masses made up of fibrous stroma and chronic inflammatory infiltrates with absence of anaplasia and mitotic figures [5]. Despite that IPTs show a benign clinical course, it has been reported that they can have mediastinal invasion, local recurrences, metastases, and sarcomatous degeneration [3].

Tracheal involvement by IPTs often lead to obstructive respiratory symptoms [2]. In the present case, the patient presented with respiratory distress and stridor.

The histopathologic appearance of IPT is characterized by myofibroblastic spindle-shaped cells in the background of an inflammatory cell infiltrate consisting of lymphocytes, plasma cells, and eosinophils. These tumors have been found to be positive for the ALK gene rearrangements [5]. Similar findings are reported in our case.

Treatment of tracheal IPTs depends on many factors such as location of the tumor, its extension, and chest and general condition of the patient. The treatment may entail steroid therapy, endoscopic laser excision, and surgical excision [3]. Endoscopic mass reduction or tracheostomy are options for inoperable cases.

The commonest surgical approach is conservative endoscopic laser resection. Bumber et al. [6] reported successful management of tracheal and subglottic pseudotumors using endoscopic CO2 and KTP laser vaporization. The authors of this work believe that laser excision should be reserved for cases without transmural extension. However, for tracheal IPT with transmural extension, treatment of choice is complete surgical resection of the involved tracheal rings [3].

Rosai–Dorfman disease (RDD) is another rare tumor like lesion characterized by a proliferation of histiocytes (macrophages) in lymph nodes. Painless cervical lymphadenopathy is the commonest presentation (87%) [4]. Extra-nodal RDD mainly affects skin (50%), central nervous system (10%), retroorbital (5%), bone (15%), retroperitoneal (5–10%) and upper respiratory tract (10–20%) [2]. Fibrohistiocytic tumors have been reported in the trachea with unclear biologic behavior, and were interpreted as malignant or benign by different authors [7].

RDD was firstly described by Destombes [8] in 1965, and later described by Rosai and Dorfman in 1969 as sinus histiocytosis with massive lymphadenopathy which was classified as non-langerhans cell histiocytosis by the Working Group of the Histiocyte Society in 1987 [9].

Only 19 cases of RDD affecting the central airways were reported. According to the extent of involvement, two types were reported: diffuse type that was noted in 8 cases, and localized type that was documented in 11 patients [2, 4]. In the current work, the involvement of the trachea was localized in the first 3 tracheal rings.

The typical presentation consists of fever, leukocytosis, and painless cervical lymphadenopathy in 87% of cases, while other lymph node involvement was 43% [4]. Intrathoracic involvement is extremely rare, (2%) including pulmonary nodules, pleural effusion, or airway involvement [4]. Airway involvement of RDD manifests most commonly as dyspnea, stridor, airway obstruction, and intractable cough, and can be mistaken for asthma. In the current study, the patient presented with stridor, and airway compromise without fever, or lymphadenopathy.

Histological hallmarks of the disease include emperipolesis, positive immunohistochemical staining with S100 protein and CD68, and negative staining with CD1a [4, 10]. In this case diagnosis was confirmed based on negative reaction to CK and positive reaction to S100 protein and CD68.

Management of tracheobronchial RDD depends on the extent of disease and the clinical presentation. Systemic steroids, sirolimus, radiotherapy, chemotherapy and immunomodulators, were recommended for RDD of diffuse and unresectable lesions [2].

Surgical excision is another option for RDD. Intraluminal growths may be tackled with endobronchial techniques such as diathermy-fulguration or snaring. In localized affection circumferential resection with end-to-end anastomosis has been utilized with excellent results. Recurrence has been reported in several cases, especially with non-surgical management. On the other hand, with surgical resection, only one patient [4] showed recurrence. Interestingly, this patient had diffuse affection of the trachea. The authors of the current work performed a successful single stage tracheal resection anastomosis with an optimum outcome a follow up period of 4 years.

In both cases of the current report, the authors adopted the same surgical principles that were described in the authors’ previous studies [11–13]. Tracheal resection anastomosis is a highly successful surgical procedure that entails resection of the diseased airway segment with primary end-to-end anastomosis. When performed by highly skilled teams, familiar with these airway surgeries, the best outcomes are always achieved [11, 14, 15].

## Conclusion

Tracheal Inflammatory myofibroblastoma, and Rosai–Dorfman diseases are rare tumor like lesions that present with upper airway obstruction. Despite being benign, these lesions may have features suggestive of malignancy, requiring prompt management. Complete surgical excision by segmental resection and primary anastomosis (if feasible) is the treatment of choice with an optimum outcome.

## Data Availability

Data are available upon reasonable request to the corresponding author.

## Abbreviations

ALK: Anaplastic lymphoma kinase
CD: Cluster of differentiation
CK: Cytokeratin
IPT: Inflammatory pseudo-tumors
KTP: Potassium titanyl phosphate
RDD: Rosai–Dorfman disease
